# Vapor Pressure, Vaping, and Corrections to Misconceptions Related to Medical Cannabis' Active Pharmaceutical Ingredients' Physical Properties and Compositions

**DOI:** 10.1089/can.2021.0173

**Published:** 2023-05-30

**Authors:** Aharon M. Eyal, Dana Berneman Zeitouni, Dor Tal, Daniel Schlesinger, Elyad M. Davidson, Noa Raz

**Affiliations:** ^1^ Bazelet Medical Cannabis Group, Or Akiva, Israel.; ^2^ Department of Anesthesiology, CCM and Pain Relief, Hadassah Hebrew University Hospital, Jerusalem, Israel.

**Keywords:** terpenes, cannabinoids, vapor pressure, boiling points, vaporization, vaping

## Abstract

Medical cannabis products contain dozens of active pharmaceutical ingredients (APIs) derived from the cannabis plant. However, their actual compositions and relative doses significantly change according to the production methods. Product compositions are strongly dependent on processing step conditions and on components' evaporation during those steps. Review of the documentation presented to caregivers and to patients show erroneous data or misinterpretation of data related to the evaporation, for example, cannabinoids' boiling points, as well as confusions between terms, such as boiling, vaporization, and evaporation. Clarifying these aspects is essential for caregivers, for researchers, and for developers of manufacturing processes. Original and literature data were analyzed, comparing composition changes during various processing steps and correlating the extent of change to components' vapor pressures at the corresponding temperature. Evaporation-related composition changes start at temperatures as low as those of drying and curing and become extensive during decarboxylation. The relative rate of components' evaporation is determined by their relative vapor pressure and monoterpenes are lost first. On vaping, terpenes are inhaled before cannabinoids do. Commercial medical cannabis products are deficient in terpenes, mainly monoterpenes, compared with the cannabis plants used to produce them. Terms, such as “whole plant” and “full spectrum,” are misleading since no product actually reflects the original cannabis plant composition. There are important implications for medical cannabis manufacturing and for the ability to make the most out of the terpene API contribution. Medical cannabis products' composition and product delivery are controlled by the relative vapor pressure of the various APIs. Quantitative data provided in this study can be used for improvement to reach better accuracy, reproducibility, and preferred medical cannabis compositions.

## Introduction

Unlike conventional drugs, most medical cannabis preparations contain multiple active pharmaceutical ingredients (APIs), including several cannabinoids and about 20 prevalent terpenes, all of which are considered to affect the results of the cannabis medical treatment. While the role of the main cannabinoids—delta-9-tetrahydrocannabinol (THC) and cannabidiol (CBD)—is studied for several decades,^1,2^ understanding the role of the terpenes in cannabis is still in its early stages.^3–10^ The focus is not only on the effects of terpenes as such (their content is relatively small), but also on synergistic modulation of the cannabinoid's functionality, referred to as the entourage effect. The extent of that effect is still debated.^5,11–16^

Many caregivers tend to think in terms of preferred strains or chemovars for treating particular indications. This also applies to extracts (“cannabis oils”) produced from such strains. Terms like “whole plant” and “full spectrum” are often used to describe such oils.^17–21^ Similarly, several cannabis clinical trials tested products characterized by strain name,^5,22,23^ rather than in terms of composition. Other commercial products, including ones used in many clinical studies, are characterized by the content of the main cannabinoids, but with no information about their terpene content.^17,19,20,24^ The Israeli regulator has specified products to be used for treating medical cannabis patients in Israel, all of which are characterized only by their content of THC, CBD, and cannabinol (CBN).^25^

This approach conflicts with a simple truth—the medical effect is determined by the doses of the main APIs in the formulation, independent of their source. The inconsistency in the compositions of the medical cannabis products reflect inconsistency in inflorescence compositions and the effects of processing conditions.^6,20,24,26–28^

The study here focuses on the changes in the composition during common steps of industrial manufacture of medical cannabis, such as drying and curing, extraction, and decarboxylation. Given the volatility of some of the cannabis APIs, mainly the monoterpenes, the following deals more specifically with vaporization of APIs, with the parameters controlling it and with their implications for the final products' composition. Some of those implications extend to API's provision during vaping. Also discussed are common misinterpretations of related physical data.

## Materials

Commercial medical cannabis extracts diluted in olive oil (“cannabis oils”) and commercial inflorescences were purchased from authorized manufacturers in Israel. Cannabinoid's standards: CBN, cannabichromene (CBC), cannabichromenic acid (CBCA), cannabigerolic acid (CBGA), cannabigerol (CBG), cannabidiolic acid (CBDA), CBD, delta-9-Tetrahydrocannabinolic acid (THCA), and THC were purchased from Cerrilliant (Cerilliant Corporation, Round Rock, TX). Cannabidivarin (CBDV), cannabidivarinic acid (CBDVA), tetrahydrocannabivarin (THCV), and delta-8-Tetrahydrocannabinol (Δ8-THC) were purchased from Restek (Bellefonte, PA).

All terpene standards were purchased from Restek (Bellefonte), Catalog No. 34095.

Ethanol for standard solution and sample preparation was of high-performance liquid chromatography (HPLC) grade (J.T. Baker).

## Methods

### Vaping trials

Commercial cannabis inflorescences were ground using a coffee grinder (five pulses of 1 sec each). About 200 mg samples of the ground material were vaporized using the Volcano Medic^®^ vaporizer (Storz and Bickel, Tuttlingen, Germany) at selected temperatures and for selected durations, all in three replicates. The inflorescence and the remaining materials following vaporization were analyzed for their cannabinoids and terpene content.

### HPLC and gas chromatography analysis

#### High-performance liquid chromatography

The analysis of cannabinoids was carried out on HPLC Waters PDA 2996, equipped with a pump, autosampler, column-oven, and a Photodiode Array detector (PDA). The analytical balance is Mettler Toledo MS205DU.

The method was developed by Bazelet and is based on HPLC reverse-phase separation with HPLC column-type C18 and UV detection. The method is fully validated for 12 cannabinoids (see in Materials section) with requirements of International Council for Harmonization of Technical Requirements for Pharmaceuticals for Human Use (ICH) guidelines,^29^ Israeli Medical Cannabis Agency (IMCA), European Pharmacopoeia (EP),^30^ and the United States Pharmacopeia (USP). The method range is 0.1–120.0% of the nominal working concentration, proved by linearity, precision, and accuracy studies. The Limit of Quantitation of the method is 0.1%. Total cannabinoids are calculated as if all of the cannabinoids are in their decarboxylated form.

#### Gas chromatography

Terpene analysis was carried out on Agilent Technologies gas chromatography (GC) system model 6890N equipped with FID detector. A CTC autosampler (Pal RTC, CTC analytics, Switzerland) was used. The column was ZB-624plus 30 m×0.25 mm×1.40 μm with He as a carrier at 1.2 mL/min constant flow.

The method, developed in Bazelet, was to determine 19 terpenes likely to be present in cannabis. The method is fully validated according to the requirements of ICH guidelines, EP and USP. The range of the method is 200–4000 ppm, proved by linearity, precision, and accuracy studies. The Limit of Quantitation of this method is 200 ppm. Due to a lack in standards, the content of all unidentified terpenes was estimated by calculating their area from α-Humulene response factor. The terpenes that are not fully identified are presented by their retention time.

## Results and Discussion

### Prevalent terpenes and their content in inflorescences and in extracts

Inflorescences of chemovars grown in Israel were analyzed for their cannabinoid and terpene contents. Table 1 presents for each terpene, ranges of concentrations, and of terpenes to total cannabinoid ratios. Table 2 presents similar data for commercial medical cannabis “oils”—cannabis extracts diluted in vegetable oils.

**Table 1.** Ranges of Terpene Concentrations and of Terpene to Total Cannabinoid Ratios in Inflorescences of *Cannabis Sativa* L. Chemovars Grown in Israel

**Group**	**Compound**	**Min (ppm)**	**Max (ppm)**	**Min (mg/g TC)**	**Max (mg/g TC)**
Monoterpenes	α-Pinene	94	5185	0.47	21.4
	Camphene	57	207	0.30	0.55
	Sabinene	260	260	1.30	1.44
	β-Pinene	121	6700	0.81	33.5
	β-Myrcene	266	9053	2.22	35.1
	δ-3-Carene	383	383		
	Carene	142	1106	1.12	7.42
	Ocimene	121	2343	0.93	5.12
	Limonene	142	3663	1.12	18.0
	p-Cymene	1885	1885		
	γ-Terpinene	101	378	0.77	2.75
	Terpinolene	201	1192	1.34	3.64
Other terpenes	Linalool	176	2020	1.21	10.1
	RT 13.0	91	697	0.54	4.08
	RT 14.7	129	373	1.00	2.66
	RT 19.0	135	1219	1.17	6.77
	Isopulegol	1439	1439		
	β-Caryophyllene	533	6778	3.44	26.8
	α-Humulene	196	7132	1.19	10.1
	Nerolidol	105	1210	0.62	6.05
	RT 20.4	61	1681	0.47	11.2
	RT 20.5	74	332	0.62	1.84
	RT 20.7	88	785	0.86	4.36
	RT 20.8	50	247	0.38	1.65
	RT 20.9	55	732	0.69	4.07
	RT 21.0	189	1446	2.06	9.64
	RT 21.1	230	2022	2.67	13.5
	Guaiol	97	1890	0.66	10.1
	Eudesmol	93	1366	0.81	7.99
	Bisabolol	117	2768	0.78	16.2
Total terpenes		3060	23,382	28.9	130

Terpenes that are not fully identified are presented by their RT. Total THC concentrations ranged up to about 25% and those of total CBD, up to 15%. Blank cells are for cases where cannabinoid concentrations are missing.

CBD, cannabidiol; RT, retention time; TC, total cannabinoids; THC, delta-9-tetrahydrocannabinol.

**Table 2.** Ranges of Terpene Concentrations and of Terpene to Total Cannabinoid Ratios in Commercial Medical Cannabis Oil Products in Israel

**Category**	**Compound**	**Min (ppm)**	**Max (ppm)**	**Min (mg/g TC)**	**Max (mg/g TC)**
Monoterpenes	β-Myrcene	219	380	0.76	2.11
	Limonene	71	323	0.34	1.3
Other terpenes	Linalool	165	653	0.57	2.92
	Isopulegol	228	242	0.92	1.29
	β-Caryophyllene	686	4833	2.37	19.4
	α-Humulene	249	1392	0.86	5.61
	Nerolidol	74	689	0.26	3.65
	RT 20.4	482	2448	3.07	9.83
	RT 20.8	202	424	1.59	1.70
	RT 20.9	338	716	2.66	2.88
	RT 21.0	726	2299	4.62	9.2
	RT 21.1	596	3028	3.8	12.2
	Guaiol	206	1725	1.33	5.95
	Eudesmol	127	437	1.00	1.76
	Bisabolol	301	2264	1.12	9.09
Total terpenes		2995	17,812	250,031	580,016

Terpenes that are not fully identified are presented by their RT. Total THC concentrations ranged up to about 20% and those of total CBD, up to 28%.

The majority of the identified terpenes belong to one of four groups—monoterpenes (hydrocarbons consisting of two isoprene units, having the molecular formula of C_10_H_16_), monoterpenoids (oxygen-containing monoterpenes, having the molecular formula of C_10_H_18_O), sesquiterpenes (hydrocarbons consisting of three isoprene units, having the molecular formula of C_15_H_24_), and sesquiterpenoids (oxygen-containing sesquiterpenes, having the molecular formula of C_15_H_26_O), see Table 3. The total inflorescence terpene content varies much between different chemovars, about 0.3% to about 2.3%, and the most prevalent ones are β-myrcene, α and β-pinene, α-humulene, and β-caryophyllene, which are in good agreement with literature results.^5,6^ Lower terpene contents were found in the commercial oils, which are particularly low in monoterpenes.

**Table 3.** Normal Boiling Points (Boiling Points at Atmospheric Pressure) and Vapor Pressure of the Various Cannabinoids and Terpenes

				**Vapor pressure (Torr)**			
**Category**	**Compound**	**Formula**	**Boiling point (°C)**	**20°C**	**49°C**	**130°C**	**180°C**
Cannabinoids	THC	C_21_H_30_O_2_	425	5.24E−07	1.38E−05	1.07E−02	1.97E−01
	CBD	C_21_H_26_O_2_	463.9	6.31E−06	1.02E−04	2.89E−02	3.45E−01
Monoterpenes	α-Pinene	C_10_H_16_	155	3.57E+00	1.65E+01	3.69E+02	1.44E+03
	Sabinene	C_10_H_16_	163	1.92E+00	9.83E+00	2.72E+02	1.17E+03
	β-Pinene	C_10_H_16_	166	2.18E+00	1.06E+01	2.66E+02	1.09E+03
	β-Myrcene	C_10_H_16_	168	1.69E+00	8.72E+00	2.43E+02	1.05E+03
	Limonene	C_10_H_16_	176	1.13E+00	6.12E+00	1.88E+02	8.47E+02
	Terpinolene	C_10_H_16_	185	7.99E−01	4.44E+00	1.44E+02	6.65E+02
Monoterpenoids	Linalool	C_10_H_18_O	198	1.15E−01	9.36E−01	6.60E+01	4.27E+02
	α-Fenchol	C_10_H_18_O	201	7.54E−02	6.63E−01	5.48E+01	3.81E+02
	α-Terpineol	C_10_H_18_O	217	3.01E−02	2.92E−01	2.93E+01	2.22E+02
Sesquiterpenes	β-Caryophyllene	C_15_H_24_	263	2.12E−02	1.70E−01	1.17E+01	7.48E+01
	α-Humulene	C_15_H_24_	276	1.00E−02	8.77E−02	7.19E+00	4.98E+01
	Selina-3,7(11)-diene	C_15_H_24_	282	1.17E−02	9.71E−02	7.10E+00	4.67E+01
Sesquiterpenoid	Guaiol	C_15_H_26_O	290	8.99E−05	1.80E−03	7.86E−01	1.13E+01
	Eudesmol	C_15_H_28_O	295	4.98E−05	9.55E−04	3.86E−01	5.37E+00
	Bisabolol	C_15_H_26_O	314	2.24E−05	5.05E−04	2.84E−01	4.57E+00

### Vaporization, boiling, boiling points, and evaporation—resolving common misconceptions

The boiling points of some of the cannabis terpenes are shown in Table 3. Unless differently specified, the term boiling point refers to the temperature of boiling at atmospheric pressure, also termed normal boiling point. Typically, the boiling points increase in the following sequence: monoterpenes, monoterpenoids, sesquiterpenes, and sesquiterpenoids.

It is well known that terpenes are volatile and that the characteristic aroma of different chemovars is determined by some of the more volatile terpenes.^31^ Also known is that some of the terpenes are lost due to vaporization during processing and that the extent of monoterpene loss is greater compared with sesquiterpenes.^32,33^ Yet, there is much erroneous data and misinterpretation of data related to the boiling points of cannabinoids and of some terpenes. For example, dozens of documents and websites report that the boiling point of THC is 155–157°C and that that of CBD is between 160°C and 180°C,^34^ while the actual (normal-) boiling points are markedly higher as detailed in the following. Similarly, the boiling point of β-caryophyllene is shown in many publications as 119°C or 130°C,^34^ while the actual boiling point is about 263°C.^35^ Also, in some cases, the terms boiling, vaporization, and evaporation are confused. These aspects are briefly discussed in this study, based on the vapor pressure property of the involved compounds.

**FIG. 1.**
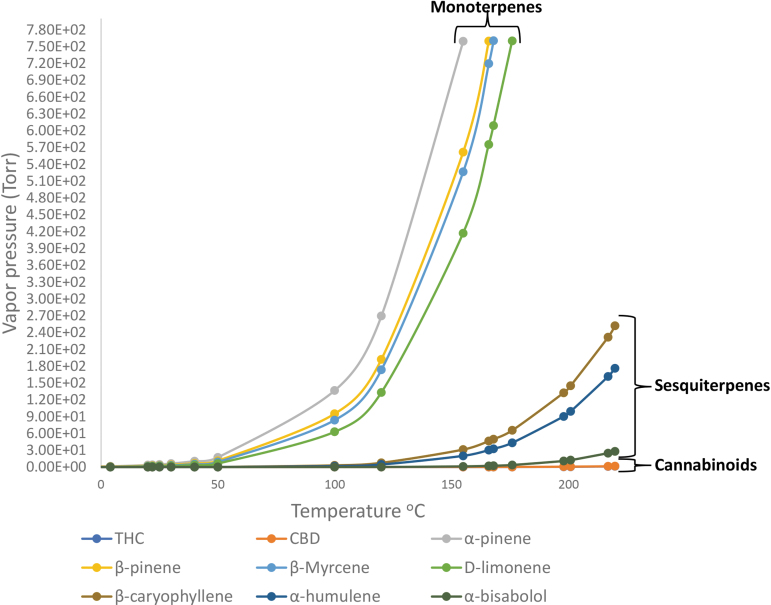
Vapor pressures of some terpenes at various temperatures, based on Clausius–Clapeyron equation. The vapor pressures of monoterpenes are markedly higher than those of sesquiterpenes and greatly higher than those of cannabinoids. The critical role of vapor pressure in determining the evaporation or preservation of a compound explains the actual composition of the various cannabis products.

The vapor pressure is the pressure exerted by a vapor in thermodynamic equilibrium with the liquid phase.^36^ At a given temperature, more volatile liquids have higher vapor pressures. The vapor pressure increases nonlinearly with the temperature, according to the Clausius–Clapeyron equation.^37^ Figure 1 presents vapor pressure dependency on temperature for some of the terpenes, based on this equation. The temperature at which the vapor pressure of a compound reaches the atmospheric pressure (760 torr, 100 KPa) is the boiling point of that compound, also referred to as the normal boiling point. At this temperature, the vapor pressure becomes sufficiently high to overcome the atmospheric pressure and to lift the liquid to form bubbles, which is referred to as boiling.

Under vacuum, lower temperatures are sufficient to bring the vapor pressure to the external one, so that at high vacuum of 0.05 torr, THC boils at about 155°C.

There is no practical direct way of measuring the temperature at which the vapor pressures of THC and CBD reach normal room pressure (about 760 torr), since at such elevated temperatures, these cannabinoids are instable and decompose. The normal boiling points can, however, be estimated by extrapolating vapor pressure at lower temperatures, using the Clausius–Clapeyron equation. For example, the vapor pressures of THC at 155°C and 190°C are about 0.05 torr and 0.3 torr, respectively, leading to boiling points higher than 400°C, in agreement with the results of McClements, Umnahanant et al., and Lovestead and Bruno.^38–40^

The term vaporization refers to any form of converting liquid to gas, including at temperatures below the boiling point (as in cannabis inflorescence drying at ambient temperature). Such vaporization below the boiling point is termed evaporation. At a given temperature, compounds with different boiling points vaporize at different rates. The relative rate of vaporization depends on the corresponding vapor pressure.

Calculated vapor pressures at various temperatures are added in Table 3. These data show that a relatively small difference in the boiling point may result in a significant difference in the vapor pressure, leading to major differences in evaporation rate of the various compounds. For example, at 20°C, the rate of α-pinene evaporation is about three times greater compared with limonene. The vapor pressure of an API is the most important factor responsible for its evaporation or preservation in the cannabis products.

The following sections deal with vaporization and evaporation of cannabinoids and terpenes at various stages of cannabis processing and administration.

### Terpene loss on drying and curing at about the ambient temperature

Harvested cannabis inflorescences typically contain about 80% moisture and goes through postharvest drying and curing. Various manufacturers adopt different drying methods, characterized among others by different treatment durations ranging from few weeks to a couple of months. Although conducted at relatively low temperatures, some terpene evaporation takes place.

Hanuš and Hod's^5^ Tables 24 and 25 present the prevalent terpenes of chemotype Pandora's Box in fresh and in dry forms, normalized to the most prevalent terpene in each case (terpinolene and β-caryophyllene, respectively). In Table 4, we have recalculated the terpene contents for both the fresh and the dried forms relatively to the content of β-caryophyllene in each. It shows that, as a result of drying, the content of monoterpenes drastically decreases, while that of sesquiterpenes is relatively kept. Out of the two main monoterpenes, β-myrcene disappears from the most prevalent terpenes, and the terpinolene/β-caryophyllene ratio drops by more than a factor of 3. For most sesquiterpenes, changes are small.

**Table 4.** The Content of the Most Prevalent Terpenes in the Fresh and in Dried Pandora's Box Inflorescences, Relative to Their β-Caryophyllene Content

**Terpene**	**Formula**	**Fresh**	**Dried**
β-Caryophyllene	C_15_H_24_	100	100
Terpinolene	C_10_H_16_	116.0	37.2
α-Humulene	C_15_H_24_	40.0	35.9
γ-Elemene	C_15_H_24_	26.2	30.8
Selina-3,7(11)-diene	C_15_H_24_	17.6	20.6
β-Myrcene	C_10_H_16_	15.1	
Germacrene B	C_15_H_24_	13.2	10.4
α-Cadinene	C_15_H_24_	12.9	
Bulnesol	C_15_H_26_O	11.6	15.4
10-epi-γ-eudesmol	C_15_H_26_O		14.7
Eudesmol	C_15_H_26_O		10.8
Guaiol	C_15_H_26_O		10.5

Recalculation of Hanuš and Hod,^5^ Data in Tables 24 and 25.

Similar observations were reported by Ross and Elsohly 1996.^41^ These results are in agreement with the calculated vapor pressures in Table 3, showing that, at ambient temperature, the vapor pressure of β-myrcene is about 80 times greater compared with β-caryophyllene.

Thus, even dried inflorescences, with no further processing, do not reflect properly the terpene content of the cannabis plant, the monoterpenes to sesquiterpene ratio, or the terpenes/cannabinoids ratios. Differently put, dried inflorescence in whatever form it was dried, is already not a “full-spectrum” composition.

### Evaporation during sterilization

A common step in cannabis processing involves sterilization or pasteurization to reduce the bacterial and fungal count. Methods used include beta- or gamma-irradiation, steam treatment, and radiofrequency treatment,^42^ some of which involves heating the inflorescence to at least about 60°C.^43^ Steam treatment also requires a follow-up drying step. Terpenes evaporate according to their vapor pressure at the corresponding temperature,^43^ unless the treatment is conducted in a sealed package capable of withstanding the heating-generated pressure.

### Terpenes and cannabinoids in extracts exposed to temperatures of up to 49°C

Sexton et al.,^31^ present data for cannabinoid and terpene content of six cannabis chemovar inflorescences and of extracts generated from their trim. Extracts were produced by extracting with supercritical CO_2_ in a closed-loop system for 6 h. The temperatures of the extractor, of the separator, and of the condenser were 43°C, 60°C, and 4°C, respectively. Extraction pressure was 1850 psi. After separating the CO_2_ in the separator, the concentrated extract was treated in a vacuum oven for 24 h at a reduced pressure and at 49°C for the removal of residual water. Additionally, the authors presented for each reported component the ratios between its concentration in the extract, on the one hand, and in the flowers, on the other.

The conclusion reached was that the extraction protocol enhances the potency of both cannabinoids and terpenoids, but in a different fashion.^31^

From Sexton et al.^31^ data, we have calculated for each terpene the terpene to total cannabinoid weight/weight ratios in both the flower (Rf) and the extract (Re). Also calculated was Rp=Re/Rf. Rp magnitude is determined mainly by two parameters: (1) extraction selectivity,^11,27,32,44^ and (2) components' loss to evaporation.

Consider first a theoretical terpene with volatility so low that no loss of it takes place during processing, so that Rp is solely determined by extraction selectivity. In such case, Rp >1 indicates higher selectivity to terpenes over cannabinoids and a relatively low cannabinoid extraction yield. For example, in case the terpene is completely extracted, Rp of 1.8 and 2.7 would correspond to cannabinoid extraction yields of 56% and 37%, respectively, which are far too low for an industrial operation.

**FIG. 2.**
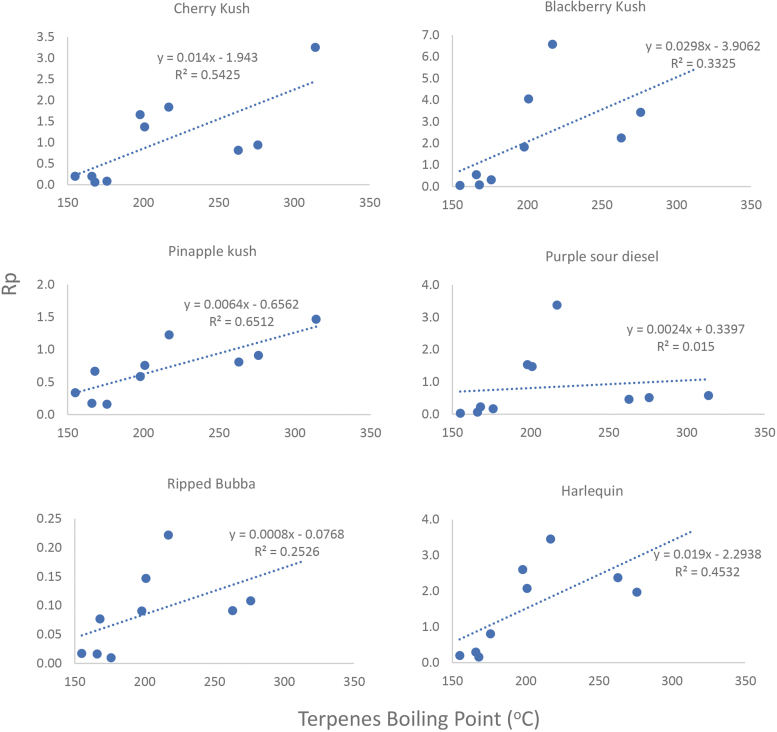
Terpene/cannabinoid ratio changes between inflorescences (Rf) and extracts (Re) (Rp=Re/Rf) as a function of terpene boiling points. Data from six cannabis chemovars' study by Sexton et al.^31^

In Figures 2, we present for each chemovar the calculated Rp values and their dependencies on the terpene boiling points. A good correlation is found, indicating that the main contributor to the difference between the extracts and the inflorescences in Sexton's work is evaporation, mostly taking place during the dewatering step. This assumption is supported by the data in Table 3, presenting the estimated vapor pressures of the various terpenes at 49°C. Accordingly, the evaporation rates of α-pinene and linalool are about 190 times and about 11 times greater, respectively, compared with α-humulene.

In summary, while solvent selectivity might play a role, if showing high preference to cannabinoids over terpenes, the change in the terpene/cannabinoid ratio in the extract compared with that of the plant material source is mainly dependent on the volatility of the terpenes, more specifically on their vapor pressure at the treatment temperature. Even at the relatively low temperature of Sexton's method, volatile terpenes are lost at a rate of 70–95%. At the same time, the nonvolatile terpenes are mostly preserved. Using different solvents with different boiling points may change the extent of removal,^45^ but not the main outcome: reduction in the extract of the overall proportion of the terpenes, in the monoterpene/cannabinoid ratio and in the monoterpene/sesquiterpene ratio.

### Terpenes and cannabinoids in decarboxylated inflorescences

According to a processing method, decarboxylation is conducted in the inflorescences before the extraction step.^6^ Romano and Hazekamp (2013)^45^ have tested two methods of decarboxylating inflorescence of the variety Bedrocan: 5 min in a water bath at 98–100°C and 30 min in an oven at 145°C. The decarboxylation efficiency of the former was small, but it still involved the loss of about one half of the main monoterpenes. Loss of sesquiterpenes at these conditions was smaller, but was nearly complete at 145°C.

Shapira et al.^7^ reported terpene content of three chemovar inflorescences before decarboxylation and after it. Decarboxylation involved keeping in an oven at 130°C for 1 h. Shapira et al.^7^ did not report the cannabinoid concentrations so that they cannot be used as the nonvolatile reference here. Instead, we have used for the calculations the content of each terpene relative to that of β-caryophyllene before decarboxylation R(C) and after it R(ADC). Table 5 presents the ratio of these ratios, Rd=R(ADC)/R(C), for Shapira's three chemovars, where Rd <1 represent higher losses during decarboxylation compared with β-caryophyllene losses.

**Table 5.** Terpenes to β-Caryophyllene Ratios [Rd=R(DAC)/R(C)] Post Decarboxylation Calculated from the Data of Shapira et al.^7^ Report

**Terpene**	**Formula**	**Rd (type I)**	**Rd (type II)**	**Rd (type III)**
α-Pinene	C_10_H_16_	0.17		0.013
Sabinene	C_10_H_16_	0.05		
β-Pinene	C_10_H_16_			
β-Myrcene	C_10_H_16_	0.04		0.05
Limonene	C_10_H_16_	0.39	0.45	0.46
Linalool	C_10_H_18_O	0.43	0.58	0.47
α-Fenchol	C_10_H_18_O	0.40	0.38	0.41
α-Terpineol	C_10_H_18_O	0.50	0.47	0.43
β-Caryophyllene	C_15_H_24_	1	1	1
α-Humulene	C_15_H_24_	1.04	1.08	0.95
Ledene	C_15_H_24_	1.04	1.09	0.81
Valencene	C_15_H_24_	1.08	1.07	0.98

Blank cells are for contents below the level of quantification.

The results clearly show that the decarboxylated flowers are depleted in monoterpenes and in monoterpenoids compared with the sesquiterpene β-caryophyllene. The extent of that depletion depends on the boiling points and on the vapor pressures at the decarboxylation temperature (130°C). According to the data in Table 3, at that temperature, the evaporation rates of β-pinene and α-terpineol are about 900 times and about 100 times greater, respectively, compared with α-bisabolol.

As indicated, these results were calculated from data for decarboxylation in the inflorescence before extraction. According to an alternative processing method, decarboxylation is conducted at a later stage—on the extract formed after solvent removal and before dilution in vegetable oil. Treatment temperature and duration are similar to those of decarboxylation in the inflorescence and so is the impact on terpene evaporation.

### Terpene and cannabinoid ingestion on using vaporizers at 180°C to 220°C

Vaporization also plays an important role in using vaporizers (“vaping”) to deliver the cannabis APIs—cannabinoids and terpenes—from cannabis inflorescence. Ground inflorescence is heated in dedicated vaporizers for few minutes and the formed vapors are inhaled. In most vaporizers, the vapors are inhaled directly, while in others, the vapors are collected in a kind of a balloon and then inhaled from it. Many forms and designs of vaporizers exist on the market, with prices ranging from a few USD to almost 1000 USD. The recommended operation temperature is typically 160–220°C, or more specifically, about 180°C. This temperature is more than 200°C under the normal boiling points of THC and CBD.

Lanz et al.^46^ have compared vaporization of cannabis in five commercial vaporizers, all of which were operated at 210°C for 3 min. Vaporization extent was 55–83% for THC and 46–70% for CBD. No data were provided for terpene vaporization. To compare terpene vaporization to that of cannabinoids, we have conducted a trial operating a Volcano vaporizer (one of the five used by Lanz et al.) at 180°C to 220°C, for durations extending from 20 to 180 sec.

Table 6 presents THCA, THC, and total THC concentrations before heating and in the residues after it. On temperature elevation, the acid form cannabinoids, for example, THCA and CBDA, decarboxylate to the neutral derivates, for example, THC and CBD. According to Table 6, at the conditions of the trial here, decarboxylation approaches completion within 40 sec at 180°C and is practically completed within 20 sec at 220°C.

**Table 6.** Concentrations and Total Amounts of THCA, THC, and Total THC Found in Ground Inflorescence Before and After Heating in a Volcano Vaporizer at Several Temperatures for Several Durations

**Temperature (°C)**	**Time (sec)**	**%THC (w/w)**	**%THCA (w/w)**	**%Total THC (w/w)**	**Total THC (mg)**	**Degree of evaporation**	***N***
Before heating		0.80±0.02	15.6±0.19	14.5±0.15	30.0±0.65	0%	6
180	20	9.77±0.92	7.57±0.78	16.4±0.37	27.2±0.12	−9%	3
	40	13.9±0.57	1.37±0.05	15.1±0.52	23.8±1.18	−21%	3
	60	13.1±0.19	0.97±0.10	14.0±0.17	23.3±0.29	−22%	3
	90	11.6±0.31	0.33±0.03	11.9±0.29	19.7±0.43	−34%	3
	120	10.3±0.21	0.50±0.24	10.7±0.32	16.8±0.47	−44%	3
	180	7.07±0.16	0.03±0.03	7.10±0.15	10.3±0.27	−66%	3
200	20	12.7±0.35	1.87±0.64	14.4±0.23	24.2±0.52	−19%	3
	40	11.2±0.35	0.27±0.07	11.4±0.41	18.3±0.84	−39%	3
Before heating		2.20±0.03	7.10±0.05	8.4±0.07	16.8±0.17	0.0%	3
220	20	6.52±0.05	0.13±0.11	6.6±0.08	11.0±0.34	−34.2%	3
	40	5.29±0.28	0.00±0.00	5.3±0.28	8.33±0.55	−50.3%	3

THCA, delta-9-tetrahydrocannabinolic acid.

Next, these neutral cannabinoids evaporate at a rate dependent on the operating temperature. Here, 34% and 50% of the THC evaporate within 20 and 40 sec, respectively, at 220°C, but only 10% and 20%, respectively, at 180°C, which is in good agreement with the corresponding vapor pressures. According to the Clausius–Clapeyron equation, compared with vaping at 160°C, the evaporation rate of THC at 180°C, 200°C, and 220°C are about 3 times, 8.4 times, and 19.4 times greater, that is, increasing by a factor of nearly 3 per 20°C of temperature elevation. Extrapolating the results in Table 6 shows that, at the conditions used here, at 180°C, more than 3 min are required for approaching complete evaporation of the THC.

**FIG. 3.**
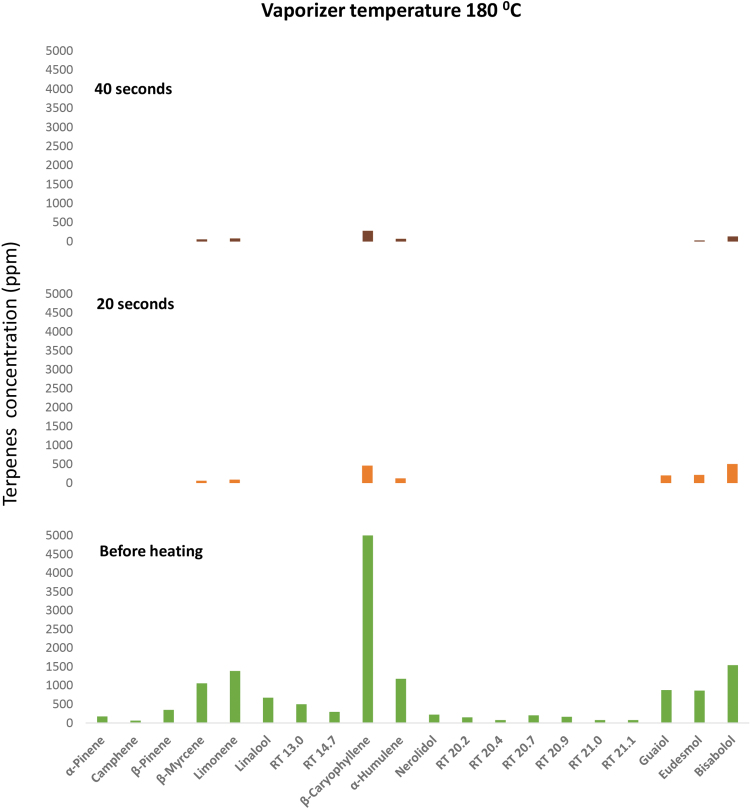
Terpene's concentration measured in *Cannabis sativa* L. inflorescence before and after treatment in a Volcano vaporizer at 180°C for 20 and 40 sec. As seen, monoterpenes almost completely evaporate following 20 sec of vaping.

Figure 3 presents the terpene content of the starting inflorescence and of that inflorescence after 20 and 40 sec at 180°C. Monoterpenes and monoterpenoids are mostly evaporated and the sesquiterpenes and sesquiterpenoids are markedly evaporated, before there is significant evaporation of THC. As can be concluded from Table 3, monoterpenes and monoterpenoids reach boiling at the temperature of vaporizer operation or approach it, whereas other terpenes have high vapor pressures, resulting in high rates of evaporation. Thus, for example, the rates of vaporization at 180°C of bisabolol (sesquiterpenoid), β-caryophyllene (sesquiterpene), linalool (monoterpenoid), and β-myrcene (monoterpene) are about 23, 380, 2200, and >3500 times greater compared with THC.

It is important to note that, while the data reported here applies to the composition of the vapors formed in the vaporizer, the composition of the inhaled vapors may differ. That is since the formed vapors cool down to some extent before being actually inhaled. On cooling, the cannabinoids (and possibly also some high-boiling terpenes) condense to small droplets. Those droplets may partially adsorb on the cooler parts of the vaporizer or stay within the balloon, when one is used. As a result, the proportion of the low-boiling terpenes in the actually inhaled vapors is even higher than that in the vaporizer-formed vapors.

These data and analysis show that, in using a vaporizer at recommended conditions, in total, the inhaled terpene to cannabinoid ratio is shifted away from that in the inflorescence. Potentially more importantly, the vast majority of the terpenes are inhaled before a significant fraction of the cannabinoids is received. It is not clear yet what the implications are of this time difference for the entourage effect.

## Conclusion

Any postharvest processing step changes the composition of the cannabis product, even if conducted at relatively low temperatures, such as in drying and curing. Industrial medical cannabis products, at least the vast majority of them, are therefore different from the harvested inflorescence and as such are not “whole plant” or “full-spectrum” ones. The administered products are depleted in terpenes compared with the inflorescence at harvest, particularly with regard to monoterpenes and monoterpenoids. Since cannabinoids and sesquiterpenes do not show marked evaporation during processing, the ratios of monoterpenes to cannabinoids and monoterpenes to sesquiterpenes drop drastically, with potential important implications for the entourage effect.

The vapor pressure changes nonlinearly with the temperature so that small variations in temperature have strong impacts. Thus, the rate of evaporation of β-myrcene during decarboxylation at 120°C is about 2.1 times greater than that at 100°C, while for THC the rate at 120°C is 4.3 times greater than that at 100°C. Uniform heating of inflorescences is practically impossible in industrial operations, processing batches of dozens of kilograms of solid material with varying bulk densities. Combined with the strong dependence of the vapor pressure on the temperature, this means that the composition of the industrial products is not uniform and is not reproducible between batches.

The normal boiling points of the cannabinoids are much higher than what is usually considered, that is, above 400°C, rather than about 160°C, as mistakenly stated in many users' publications. Yet, cannabinoids' evaporation at the temperatures of vaporizer operation is feasible. It is important to note however, that terpenes, particularly monoterpenes, are mostly inhaled before the cannabinoids are.

These findings and analyses have important implications for producers, caregivers, and users of medical cannabis in the form of cigarettes or through vaporizer. There is much room for improvement to reach accuracy, reproducibility, and preferred medical cannabis compositions, making the most of the entourage effect. The analysis in this study provides tools for reaching such improvements.

## Authors' Confirmation Statement

All authors have read and agreed to the published version of the article.

## Abbreviations Used

Δ8-THC: delta-8-tetrahydrocannabinol
APIs: active pharmaceutical ingredients
CBC: cannabichromene
CBCA: cannabichromenic acid
CBD: cannabidiol
CBDA: cannabidiolic acid
CBDV: cannabidivarin
CBDVA: cannabidivarinic acid
CBG: cannabigerol
CBGA: cannabigerolic acid
CBN: cannabinol
EP: European Pharmacopoeia
HPLC: high-performance liquid chromatography
IMCA: Israeli Medical Cannabis Agency
PDA: Photodiode Array detector
RT: retention time
TC: total cannabinoids
THC: delta-9-tetrahydrocannabinol
THCA: delta-9-tetrahydrocannabinolic acid
THCV: tetrahydrocannabivarin
USP: United States Pharmacopeia

## References

[1] ElSohly MA, Chandra S, Radwan M, et al. A comprehensive review of Cannabis Potency in the United States in the last decade. Biol Psychiatry Cogn Neurosci Neuroimaging. 2021;6:603–606. 33508497 10.1016/j.bpsc.2020.12.016

[2] Pertwee RG. Cannabinoid pharmacology: the first 66 years. Br J Pharmacol. 2006;147 Suppl 1(Suppl 1):S163–S171. 16402100 10.1038/sj.bjp.0706406 PMC1760722

[3] Mechoulam R, Hanuš LO, Pertwee R, et al. Early phytocannabinoid chemistry to endocannabinoids and beyond. Nat Rev Neurosci. 2014;15:757–764. 25315390 10.1038/nrn3811

[4] Hanuš LO, Meyer SM, Muñoz E, et al. Phytocannabinoids: a unified critical inventory. Nat Prod Rep. 2016;33:1357–1392. 27722705 10.1039/c6np00074f

[5] Hanuš LO, Hod Y. Terpenes/terpenoids in Cannabis: are they important? Med Cannabis Cannabinoids. 2020;3:25–60. 34676339 10.1159/000509733 PMC8489319

[6] Ternelli M, Brighenti V, Anceschi L, et al. Innovative methods for the preparation of medical Cannabis oils with a high content of both cannabinoids and terpenes. J Pharm Biomed Anal. 2020;186:113296. 32334134 10.1016/j.jpba.2020.113296

[7] Shapira A, Berman P, Futoran K, et al. Tandem mass spectrometric quantification of 93 terpenoids in Cannabis using static headspace injections. Anal Chem. 2019;91:11425–11432. 31369251 10.1021/acs.analchem.9b02844

[8] Russo EB, Marcu J. Cannabis pharmacology: the usual suspects and a few promising leads. Adv Pharmacol. 2017;80:67–134. 28826544 10.1016/bs.apha.2017.03.004

[9] Russo EB. The case for the entourage effect and conventional breeding of clinical cannabis: no “Strain,” no gain. Front Plant Sci. 2019;9:1–8. 10.3389/fpls.2018.01969 PMC6334252 30687364

[10] Russo EB. Taming THC: potential cannabis synergy and phytocannabinoid-terpenoid entourage effects. Br J Pharmacol. 2011;163:1344–1364. 21749363 10.1111/j.1476-5381.2011.01238.x PMC3165946

[11] Namdar D, Voet H, Ajjampura V, et al. Terpenoids and phytocannabinoids co-produced in *Cannabis sativa* strains show specific interaction for ell cytotoxic activity. Molecules. 2019;24:3031. 31438532 10.3390/molecules24173031 PMC6749504

[12] Kamal BS, Kamal F, Lantela DE. Cannabis and the anxiety of fragmentation—a systems approach for finding an anxiolytic Cannabis chemotype. Front Neurosci. 2018;12:730. 30405331 10.3389/fnins.2018.00730 PMC6204402

[13] Nuutinen T. Medicinal properties of terpenes found in *Cannabis sativa* and Humulus lupulus. Eur J Med Chem. 2018;157:198–228. 30096653 10.1016/j.ejmech.2018.07.076

[14] Santiago M, Sachdev S, Arnold JC, et al. Absence of entourage: terpenoids commonly found in *Cannabis sativa* do not modulate the functional activity of D 9-THC at human CB 1 and CB 2 receptors. Cannabis Cannabinoid Res. 2019;4:165–176. 31559333 10.1089/can.2019.0016 PMC6757242

[15] Koltai H, Namdar D. Cannabis phytomolecule “Entourage”: from domestication to medical use. Trends Plant Sci. 2020;25:976–984. 32417167 10.1016/j.tplants.2020.04.007

[16] LaVigne JE, Hecksel R, Keresztes A, et al. *Cannabis sativa* terpenes are cannabimimetic and selectively enhance cannabinoid activity. Sci Rep. 2021;11:1–15. 33859287 10.1038/s41598-021-87740-8 PMC8050080

[17] Aran A, Harel M, Cassuto H, et al. Cannabinoid treatment for autism: a proof-of-concept randomized trial. Mol Autism. 2021;12:1–11. 33536055 10.1186/s13229-021-00420-2 PMC7860205

[18] Marinotti O, Sarill M. Differentiating full-spectrum hemp extracts from CBD isolates: implications for policy, safety and science. J Diet Suppl. 2020;17:517–526. 32543253 10.1080/19390211.2020.1776806

[19] Maayah ZH, Takahara S, Ferdaoussi M, et al. The anti-inflammatory and analgesic effects of formulated full-spectrum cannabis extract in the treatment of neuropathic pain associated with multiple sclerosis. Inflamm Res. 2020;69:549–558. 32239248 10.1007/s00011-020-01341-1

[20] Nahler G, Jones TM, Russo EB. Cannabidiol and contributions of major hemp phytocompounds to the “Entourage Effect”; possible mechanisms. Altern Complement Integr Med. 2019;5:1–16.

[21] Maayah ZH, Takahara S, Ferdaoussi M, et al. The molecular mechanisms that underpin the biological benefits of full-spectrum cannabis extract in the treatment of neuropathic pain and inflammation. Biochim Biophys Acta Mol Basis Dis. 2020;1866:165771. 32201189 10.1016/j.bbadis.2020.165771

[22] Gallily R, Yekhtin Z. Avidekel Cannabis extracts and cannabidiol are as efficient as Copaxone in suppressing EAE in SJL/J mice. Inflammopharmacology. 2019;27:167–173. 30291491 10.1007/s10787-018-0536-3

[23] Baron EP, Lucas P, Eades J, et al. Patterns of medicinal cannabis use, strain analysis, and substitution effect among patients with migraine, headache, arthritis, and chronic pain in a medicinal cannabis cohort. J Headache Pain. 2018;19:37. 29797104 10.1186/s10194-018-0862-2 PMC5968020

[24] Potter DJ. A review of the cultivation and processing of cannabis (*Cannabis sativa* L.) for production of prescription medicines in the UK. Drug Test Anal. 2014;6:31–38. 10.1002/dta.1531 24115748

[25] Landschaft Y, Albo B, Mechoulam R, et al. Medical Cannabis, 3rd ed. Israeli Medical Cannabis Agency, Ministry of health: Israel, 2019. https://www.health.gov.il/hozer/mmk154_2016.pdf

[26] Vitetta L, Sikali JF. Comment on: patient-reported outcomes in those consuming medical cannabis: a prospective longitudinal observational study in patients with chronic pain. Can J Anesth. 2021;68:633–644. 34350557 10.1007/s12630-021-02078-z

[27] Namdar D, Mazuz M, Ion A, et al. Variation in the compositions of cannabinoid and terpenoids in *Cannabis sativa* derived from inflorescence position along the stem and extraction methods. Ind Crops Prod. 2018;113:376–382.

[28] Lazarjani MP, Young O, Kebede L, et al. Processing and extraction methods of medicinal cannabis: a narrative review. J Cannabis Res. 2021;3:32. 34281626 10.1186/s42238-021-00087-9 PMC8290527

[29] Guideline ICH. International Council for Harmonisation of Technical Requirements for Pharmaceuticals for Human Use. Geneva, Switzerland, 2019.

[30] Europe Council of Commission. EP, Healthcare. ED for the Q of M &. European pharmacopoeia. Strasbourg, France: Council of Europe: European Directorate for the Quality of Medicines and Healthcare, 2010.

[31] Sexton M, Shelton K, Haley P, et al. Evaluation of cannabinoid and terpenoid content: cannabis flower compared to supercritical CO_2_ concentrate. Planta Med. 2018;84:E3. 28926863 10.1055/s-0043-119361

[32] Milay L, Berman P, Shapira A, et al. Metabolic profiling of cannabis secondary metabolites for evaluation of optimal postharvest storage conditions. Front Plant Sci. 2020;11:1–15. 33178249 10.3389/fpls.2020.583605 PMC7593247

[33] Casano S, Grassi G, Martini V, et al. Variations in terpene profiles of different strains of *Cannabis sativa* L. Acta Hortic. 2011;925:115–122.

[34] McPartland JM, Russo EB. Cannabis and Cannabis extracts: greater than the sum of their parts? Cannabis Ther HIV/AIDS. 2012;1:103–132.

[35] Sharma C, Al Kaabi JM, Nurulain SM, et al. Polypharmacological properties and therapeutic potential of β-caryophyllene: a dietary phytocannabinoid of pharmaceutical promise. Curr Pharm Des. 2016;22:3237–3264. 26965491 10.2174/1381612822666160311115226

[36] Fulem M, Vlastimil R. Vapor pressure of organic compounds. Measurement and Correlation, Chemisty, 2008.

[37] Salzman WR. Clapeyron and Clausius-Clapeyron equations. Tucson: Chem Thermodyn Univ Arizona, 2001.

[38] Lovestead TM, Bruno TJ. Determination of cannabinoid vapor pressures to aid in vapor phase detection of intoxication. Forensic Chem. 2017;5:79–85. 29266138 10.1016/j.forc.2017.06.003 PMC5733806

[39] McClements DJ. Enhancing efficacy, performance, and reliability of Cannabis edibles: insights from lipid bioavailability studies. Annu Rev Food Sci Technol. 2020;11:45–70. 31905012 10.1146/annurev-food-032519-051834

[40] Umnahanant P, Zafar A, Kankala V, et al. Vapor pressure and vaporization enthalpy studies of (+)-longifolene, (−)-isolongifolene and B-myrcene by correlation gas chromatography. J Chem Thermodyn. 2019;131:583–591.

[41] Ross SA, Elsohly MA. The volatile oil composition of fresh and air-dried buds of *Cannabis sativa*. J Nat Prod. 1996;59:49–51. 8984153 10.1021/np960004a

[42] Jerushalmi S, Maymon M, Dombrovsky A, et al. Effects of cold plasma, gamma and e-beam irradiations on reduction of fungal colony forming unit levels in medical cannabis inflorescences. J Cannabis Res. 2020;2:0–11. 10.1186/s42238-020-00020-6 PMC7819314 33526086

[43] Jerushalmi S, Maymon M, Dombrovsky A, et al. Effects of steam sterilization on reduction of fungal colony forming units, cannabinoids and terpene levels in medical cannabis inflorescences. Sci Rep. 2021;11:1–13. 34234177 10.1038/s41598-021-93264-y PMC8263730

[44] Moreno-Sanz G, Vera CF, Sánchez-Carnerero C, et al. Biological activity of *Cannabis sativa* L. extracts critically depends on solvent polarity and decarboxylation. Separations. 2020;7:1–16.

[45] Romano LL, Hazekamp A. Cannabis Oil: chemical evaluation of an upcoming cannabis-based medicine. Cannabinoids. 2013;1:1–11.

[46] Lanz C, Mattsson J, Soydaner U, et al. Medicinal Cannabis: in vitro validation of vaporizers for the smoke-free inhalation of Cannabis. PLoS One. 2016;11:e0147286. 26784441 10.1371/journal.pone.0147286 PMC4718604

